# Low-Temperature Fabrication of IZO Thin Film for Flexible Transistors

**DOI:** 10.3390/nano11102552

**Published:** 2021-09-29

**Authors:** Xingwei Ding, Bing Yang, Haiyang Xu, Jie Qi, Xifeng Li, Jianhua Zhang

**Affiliations:** 1 Key Laboratory of Advanced Display and System Application, Ministry of Education, Shanghai University, Shanghai 200072, China; xwding@163.com (X.D.); hyxu@shu.edu.cn (H.X.); jhzhang@oa.shu.edu.cn (J.Z.); 2 School of Mechatronics and Automation, Shanghai University, Shanghai 200072, China; 3 School of Microelectronics, Shanghai University, Shanghai 200072, China; byang@shu.edu.cn; 4 Research and Development Department, Air Liquide Innovation Campus Shanghai, Shanghai 201108, China; jie.qi@airliquide.com

**Keywords:** thin film transistors (TFTs), flexible, low-temperature, ultraviolet (UV)

## Abstract

Solution-processed thin film transistors (TFTs) used in flexible electronics require them to be fabricated under low temperature. Ultraviolet (UV) treatment is an effective method to transform the solution precursors into dense semiconductor films. In our work, high-quality indium zinc oxide (IZO) thin films were prepared from nitrate-based precursors after UV treatment at room temperature. After UV treatment, the structure of IZO thin films was gradually rearranged, resulting in good M–O–M network formation and bonds. TFTs using IZO as a channel layer were also fabricated on Si and Polyimide (PI) substrate. The field effect mobility, threshold voltage (*V*_th_), and subthreshold swing (*SS*) for rigid and flexible IZO TFTs are 14.3 and 9.5 cm^2^/Vs, 1.1 and 1.7 V, and 0.13 and 0.15 V/dec., respectively. This low-temperature processed route will definitely contribute to flexible electronics fabrication.

## 1. Introduction

Recently, metal oxide semiconductors used as channel layers in thin film transistors (TFTs) have been extensively investigated due to their good transparency and electronic conductivity [1,2,3]. Increasing demand for next-generation flexible electronics has resulted in increased attention on applications with high performance and low-cost materials and processes. Although TFTs fabricated by conventional vacuum-based methods have advantages, their high cost and area-limited uniformity restrict their application (requiring long processing times in high vacuum environments for successful film deposition). Therefore, the solution-based process shows great potential for next-generation devices owing to its cost-effective fabrication, large-area deposition, and simple manufacturing process [4,5,6,7,8].

To achieve high densification, sol–gel thin films require a very high temperature, which provides enough thermal energy to remove impurities and form a metal–oxygen–metal (M–O–M) structure [9,10]. This temperature is usually higher than 300 °C. Such a high processing temperature is not suitable for flexible polymer substrate. Therefore, exploring a low-temperature solution method is necessary.

Sung Kyu Park et al. provided a new theoretical way to fabricate TFT at room temperature [11]. UV treatment induces the efficient densification of metal oxide thin films without additional high temperature processing. There are two stages after UV exposure for thin films: firstly, rapid chemical condensation, followed by gradual structural reorganization and densification [10]. Subsequently, a high-quality M–O–M network is formed [8]. Thus, UV treatment is an effective way to fabricate metal oxide thin films at low temperature [12]. However, the mobility is not satisfactory due to the low dielectric constant of Al_2_O_3_, which is used as a dielectric layer in TFT devices.

In this work, solution-processed high-performance IZO thin film transistors were fabricated by UV treatment. The IZO thin film was analyzed by spectroscopy. Additionally, IZO TFTs were also studied in detail. After UV exposure, IZO thin film showed a smooth surface morphology with a roughness of 0.42 nm. C-related and N-related groups were obviously decomposed, indicating the formation of high-purity thin film. Furthermore, TFTs using IZO as a channel layer and HfO_2_ as a dielectric layer were also fabricated on rigid and flexible substrates. The flexible IZO TFT showed good performance and repeatability, such as an on/off ratio of 10^7^, a mobility of 9.5 cm^2^/Vs, and an SS of 0.15 V/dec. This new process of fabricating IZO TFTs at low temperature creates insight into the applications of flexible and transparent devices.

## 2. Materials and Methods

### 2.1. Precursor Synthesis

IZO solution was prepared by dissolving indium nitrate hydrate (In(NO_3_)_3_·xH_2_O) and zinc nitrate hydrate (Zn(NO_3_)_3_·6H_2_O) in 2-methoxyethanol (2-ME). The In:Zn ratio was 7:3. The solution was stirred at 70 °C for 2 h and then stirred at room temperature for 12 h. Before use, the precursor solution was filtered through a 0.2 mm PTFE syringe filter.

### 2.2. Film Fabrication and Characterization

The IZO precursor solution was spin coated on Si substrate at 500 rpm for 5 s, followed by 3000 rpm for 30 s. The substrate was precleaned with acetone, alcohol, and deionized water sequentially in an ultrasonicator for 10 min, then dried by N_2_ flow. To densify the IZO thin film, the sample was cured under a high-pressure mercury UV lamp for 20 min under N_2_ purging. In our work, thin film transistors were fabricated using IZO as the channel layer and HfO_2_ as the gate insulator. Using atomic layer deposition (ALD), 50 nm HfO_2_ was grown at 200 °C. We also explored flexible thin film transistors using PI as a substrate and ALD-Al_2_O_3_ as a buffer layer. Al films deposited by thermal evaporation were used as a source/drain electrode of TFTs with channel widths (*W*) = 1000 µm and channel lengths (*L*) = 100 µm.

### 2.3. Characterizations

The optical transmission was carried out by a double-beam spectrophotometer (U-3900, U-3900, Hitachi, Ltd., Tokyo, Japan). The surface morphology was measured by an atomic force microscope (AFM; nanonaviSPA-400 SPM, SII Nano Technology Inc., Chiba, Japan). The AFM measurement mode used was the tapping mode. The parameters of the AFM tip (Tap150AL-G, Innovative Solutions Bulgaria Ltd., Sofia, Bulgaria) were resonant frequency: 150 KHz and force constant: 5 N/m. The measurement geometry was rectangle and the acquisition time was 4 min. Fourier Transform Infrared Spectroscopy (FTIR) was carried out by the Nicolet 5700. The chemical composition of the thin film was analyzed by X-ray photoelectron spectroscopy (XPS, Thermo Scientific K-Alpha+, Thermo Fisher Scientific Inc., Waltham, MA, USA). The crystal structure of the thin film was investigated by X-ray diffraction (XRD, Rigaku D/max-rB, Rigaku Corporation, Tokyo, Japan). The electrical properties were measured by a semiconductor parameter analyzer (Keithley 4200, Tektronix Inc., Beaverton, OR, USA).

The field-effect mobility (*μ*) and *SS* were extracted by using the following equations [13]

(1) $$I_{D}=(\frac{W}{2L}C_{i}\mu ){\left(V_{G}-V_{\mathrm{th}}\right)}^{2}$$

(2) $$SS=\frac{dV_{G}}{d(LogI_{D})}$$

where *W* and *L* are the channel width and length, respectively. *C_i_* is the capacitance per unit area of the insulator; *V_th_* is the threshold voltage; and *V_G_* is the gate voltage.

## 3. Results and Discussion

**Figure 1:**
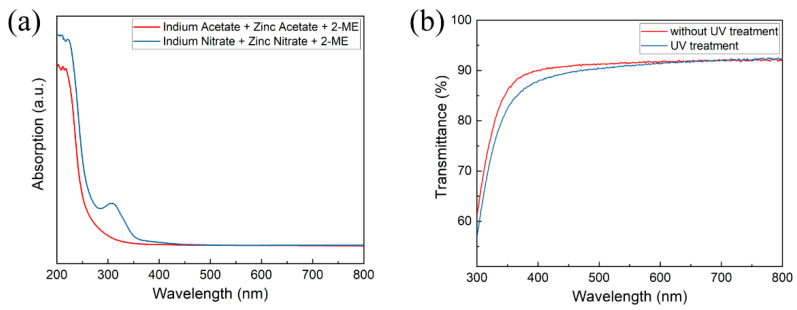
(**a**) The absorption spectra of different precursors solutions. (**b**) The transmittance of IZO thin films with and without UV treatment, respectively.

The absorption spectra of IZO thin films fabricated by acetate-based precursors and nitrate-based precursors are shown in Figure 1a. The nitrate-based precursor was chosen due to its higher UV absorptivity. The transmittance of IZO thin films on quartz glass substrates was employed to evaluate the optical performance, as shown in Figure 1b. The transparency of IZO thin films was about 90% in the visible range, favoring their application in transparent electronic devices. An interesting observation is that after UV treatment, the transparency of IZO thin film was improved in the short wavelength.

**Figure 2:**
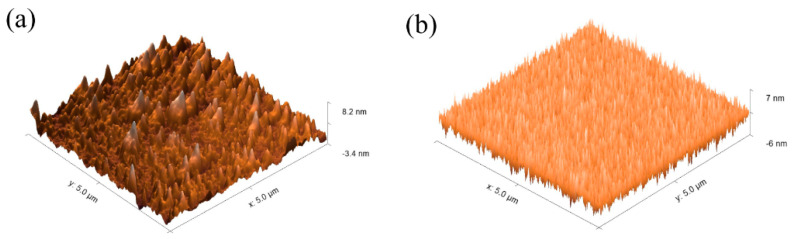
AFM images of thin films: (**a**) IZO without UV treatment, (**b**) IZO with UV treatment.

The surface morphology of thin films was characterized by AFM, as shown in Figure 2. The scanning area was 5 µm × 5 µm. It was found that IZO with UV treatment shows a small root-mean-squared (RMS) value of 0.42 nm and is free of obvious holes. On the contrary, the IZO thin film without UV treatment has a much higher RMS of 2.31 nm. After UV treatment, the densification of the IZO thin film was enhanced significantly. A good interface between the electrode and semiconductor is one of the important parameters to ensure high-performance thin film transistor devices [14,15].

**Figure 3:**
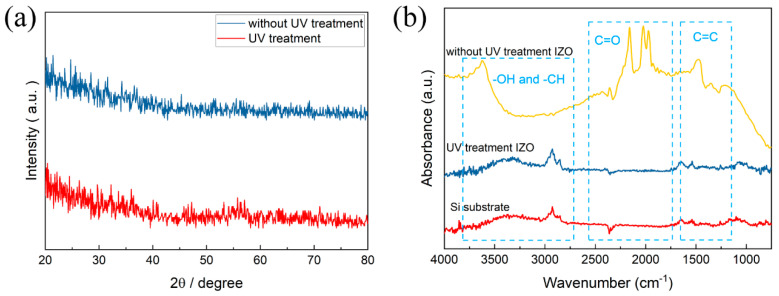
(**a**) XRD patterns and (**b**) IR spectra of IZO thin films.

The XRD patterns of IZO thin films are presented in Figure 3a. There are no obvious peaks in this pattern, indicating the amorphous structure for thin films. The amorphous phase can contribute to the formation of the semiconductor layer with a smooth surface and excellent uniformity, which is beneficial for the fabrication of TFT devices. The results are consistent with the AFM analysis.

In order to confirm the effect of UV treatment on the organic group of IZO thin films, the IR spectra were measured, as shown in Figure 3b. According to the group theory, the peaks between 2500 cm^−1^ to 4000 cm^−1^ are related to –OH and –CH stretching vibrations. The –OH vibration peak shifted to a high wavenumber after UV treatment, indicating that the free state of M–OH bonding was decomposed by the UV/O_3_ treatment [16]. The peaks at approximately 2000 cm^−1^ were related to asymmetric C=O bonding, which promotes the condensation of oxide gel films by chelating with coordination bonding to the metal elements [17]. In addition, the peaks at about 1500 cm^−1^ were associated with the vibration of C=C bonding. After UV treatment, the IR spectra for IZO thin film was found to be similar with the Si substrate. This result demonstrates that UV treatment can effectively reduce the carbon-related organic group and form high-purity thin films.

**Figure 4:**
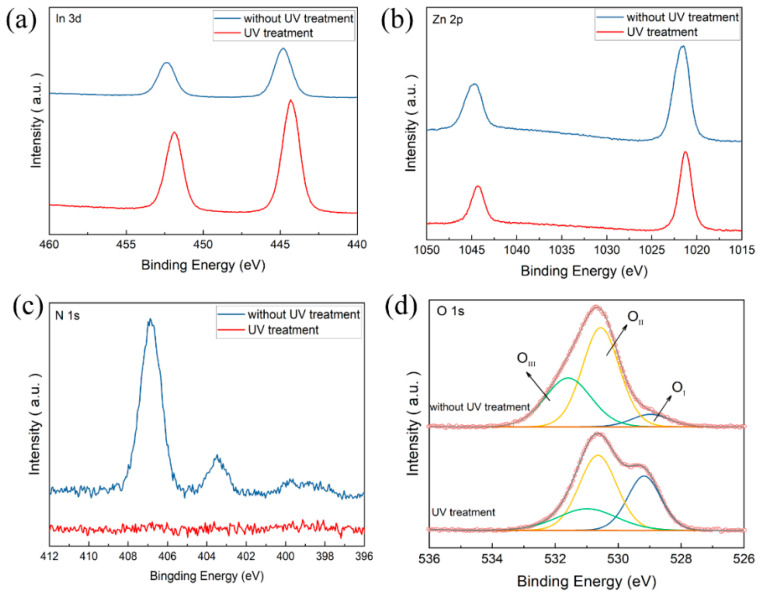
XPS spectra of (**a**) In 3d, (**b**) Zn 3d, (**c)** N 1s, and (**d**) O 1s.

To further investigate the chemical composition of IZO thin films, XPS characterization was carried out (Figure 4). All peaks were calibrated to C 1s (284.5 eV). The corresponding In and Zn high-resolution scans are shown in Figure 4a,b, respectively. Figure 4a shows that there are two related peaks near 444 cm^−1^ and 452 cm^−2^, respectively. Additionally, the peaks of Zn 2p are at approximately 1021 cm^−1^ and 1044 cm^−1^, respectively. In XPS N 1s narrow scans (Figure 4c), the N signal was not observed for IZO thin film after UV treatment, indicating the complete decomposition of the N-related group.

As shown in Figure 4d, The O 1s spectra could be fitted by three component peaks: O_I_ (528.7 eV) is related to oxygen ions (O^2−^) combined with metal cations in IZO thin film, O_II_ (530.6 eV) is associated with the oxygen vacancy, and O_III_ (531.5 eV) is attributed to bonded oxygen, such as H_2_O or O_2_ [18].The relative area of O_I_ is O_I_/O, where O = O_I_ + O_II_ + O_III_. After UV treatment, the ratio of O_I_ peak increases from 5% to 31%. This suggests that UV treatment can enhance the formation of M–O bonding in the gel films effectively [19,20].

**Figure 5:**
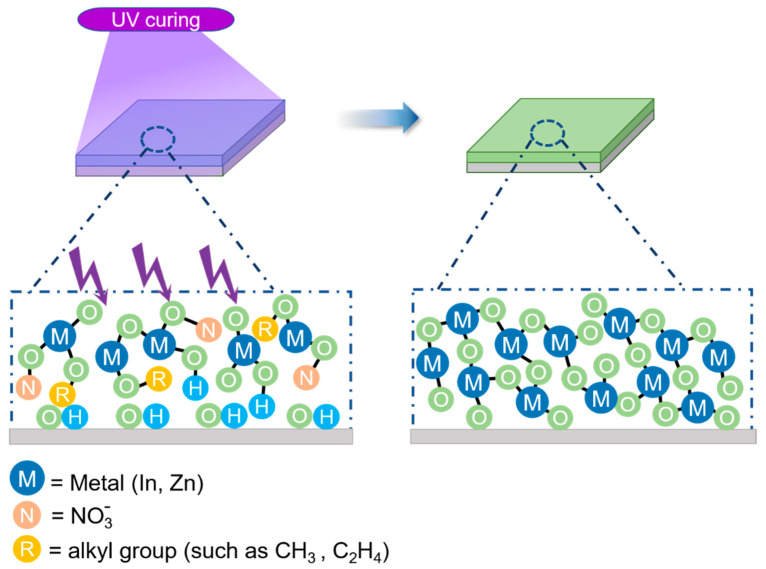
The condensation mechanism of IZO thin films by UV treatment.

The mechanism of UV-treated IZO formation is shown in Figure 5. The nitrate-based precursor exhibits high UV absorptivity. The original IZO solution is in disorder, leading to slower densification of thin films. UV treatment can lower the activation barrier for M–O bond-forming condensation, resulting in very fast film densification. The organic impurities are removed, and trap defects are greatly reduced. The M–O–M networks are reorganized without high-temperature annealing. Subsequently, a high-density M–O–M framework is formed [20,21].

**Figure 6:**
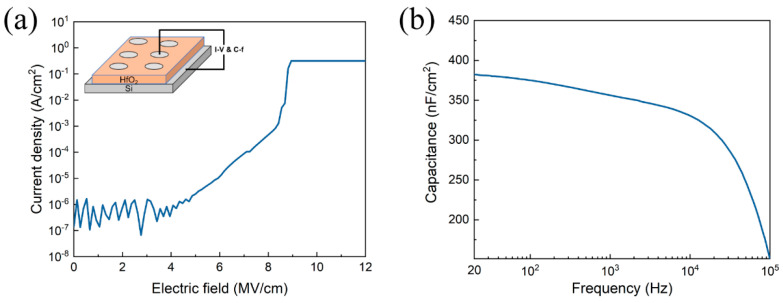
(**a**) The leakage current density and (**b**) capacitance–frequency characteristics of HfO_2_ dielectric. Inset: the schematic of the capacitor.

Before the fabrication of TFT devices, the electrical property of the HfO_2_ dielectric was evaluated, as shown in Figure 6. Figure 6a shows the leakage current density of the HfO_2_ thin film. Additionally, inset is the schematic of the capacitor. The HfO_2_ dielectric represents a low leakage current density of 10^−6^ A/cm^2^ at 2 MV/cm. In addition, the breakdown field is as high as 9 MV/cm. Figure 6b shows the areal capacitance as a function of frequency for HfO_2_ capacitor. The areal capacitance for HfO_2_ is 380 nF/cm^2^ at 20 Hz. Youn Sang Kim et al. discussed the relationship between field effect mobility and capacitance [22]. In oxide semiconductors, although the overlap of the spherical S-orbital of the metal ion gives an efficient path for carrier transport, the electron transport is governed by dense localized states between the energy bandgap. The HfO_2_ dielectric represents a very high capacitance and a strong electric field. When the HfO_2_ was used as a gate dielectric in TFT devices, abundant electrons quickly filled the upper-lying localized states, and the electrons jumped into the transport band easily, leading to the high mobility of TFTs.

This theory can be summarized as Equation (3) [20]:

(3) $$\mu ≅\mu^{0}{\left(\frac{C_{i}}{C_{i}^{0}}\right)}^{\gamma -2}$$

where *μ*^0^ is the field effect mobility at *C*_i_ = $C_{i}^{0}$ and *γ* is a constant related to the material. Thus, device mobility can be enhanced by a high-capacitance dielectric.

**Figure 7:**
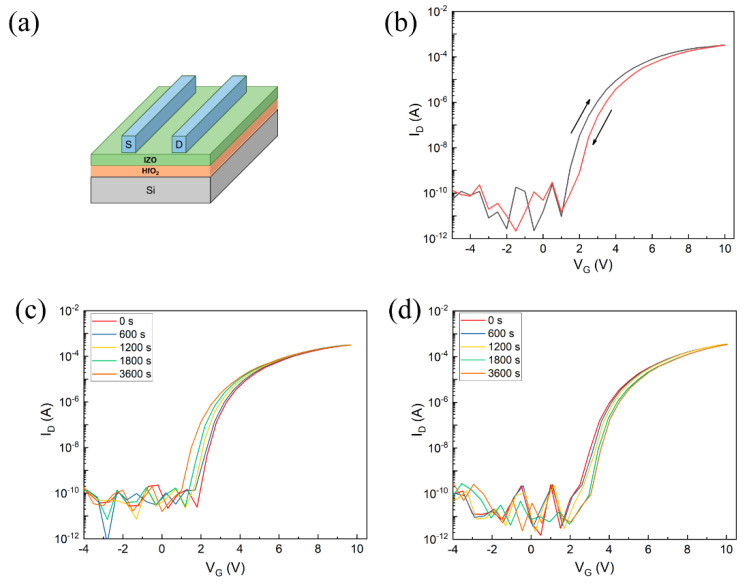
(**a**) Schematic structure of TFT device. (**b**) Transfer curve of TFT. (**c**) PBS (+10 V) and (**d**) NBS (−10 V) test for TFTs.

To study the electrical properties of IZO thin films with UV treatment, TFT was fabricated using IZO as the channel layer and HfO_2_ as the gate dielectric, as shown in Figure 7a. The transfer curves are illustrated in Figure 7b. IZO TFT exhibits typical n-type characteristics: a high on/off ratio of 10^7^, a good mobility of 14.3 cm^2^/Vs, a small *SS* of 0.13 V/dec., and a *V*_th_ of 1.1 V. It is noted that the hysteresis of transfer curve is negligible, indicating a good interface between the channel layer and dielectric layer in the TFT device [23]. The electrical performance of UV-treated IZO TFT are comparable to those of the thermally annealed devices at a high temperature in air [12,24,25]. What calls for special attention is that the IZO thin films were treated by the UV treatment system under N_2_ purging. Sung Kyu Park et al. discussed differences between metal oxide thin films annealing in N_2_ and air [26]. In the air, the photoactivation efficiency from the mercury lamp is significantly attenuated, mainly owing to absorption by molecular oxygen. Therefore, the energy is insufficient for metal alkoxides and results in poor densification, leading to inactive TFT operation.

Figure 7c,d show the positive bias stress (PBS, +10 V) and negative bias stress (NBS, −10 V), respectively. *V*_th_ shifts (∆*V*_th_) of 1.12 and 0.86 V are recorded after PBS and NBS testing for 3600 s, which is comparable with parameters from the literature [27,28,29,30,31]. The negligible ∆*V*_th_ under bias stress is due to charge trapping at the gate dielectric near the interface [30]. Furthermore, we also calculated trapping states (*N*_trap_) by [32]:

(4) $$SS=\frac{k_{B}Tln10}{q}\left[1+\frac{q^{2}}{C_{ox}}N_{\mathrm{trap}}\right]$$

where *k*_B_ is Boltzmann’s constant, *T* is the temperature in Kelvin, and *q* is the electron charge. The *N*_trap_ is 1.82 × 10^11^ eV^−1^cm^−2^ for IZO TFT, which is lower than previous reports [16,22,23,24]. The low *N*_trap_ corresponds with negligible hysteresis. Table 1 summarizes the performance parameters of various methods for the fabrication process of IZO TFTs. The electrical performance of IZO TFTs fabricated at room temperature are comparable to those of the thermally annealed devices.

**Figure 8:**
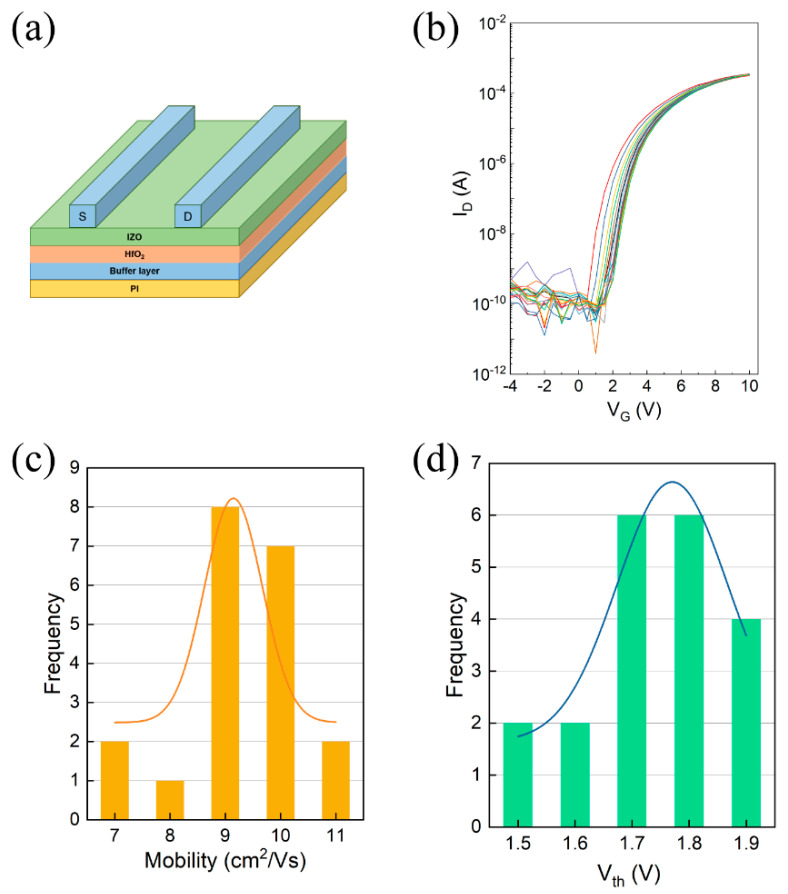
(**a**) The schematic of flexible TFT device. (**b**) Transfer curves of 20 flexible TFTs. Histogram of (**c**) mobility and (**d**) *V*_th_ for 20 devices.

Inspired by the good performance of TFTs based on Si substrate, flexible IZO TFTs were investigated on PI substrate using Al_2_O_3_ as the buffer layer, as shown in Figure 8a. We fabricated 20 IZO TFT devices. The transfer curves for 20 TFT devices are shown in Figure 8b. From Figure 8c,d, these TFT devices show outstanding uniformity and reproducibility: they have a good average mobility of approximately 9.5 cm^2^/Vs, a *V*_th_ of 1.7 V, and an *SS* of 0.15 V/dec., which is acceptable compared to previously reported devices. This result suggests that IZO with UV treatment is an ideal optional semiconductor material for flexible transparent electronics.

## 4. Conclusions

In this work, IZO thin film and related TFT devices were fabricated by UV treatment at room temperature. After UV treatment, the IZO thin film showed a lower RMS value of 0.42 nm. Oxygen ions combined with metal cations increased from 5% to 31%, indicating that UV treatment can enhance the formation of M–O bonding in the gel films effectively. After the successful fabrication of high-performance IZO TFT based on Si substrate, the flexible TFT with PI substrate was further integrated. The flexible IZO TFT showed good performance parameters and repeatability, such as an on/off ratio of 10^7^, a mobility of 9.5 cm^2^/Vs, and an *SS* of 0.15 V/dec. In a word, such a low-temperature fabricated semiconductor material offers a simple and useful approach for the exploration of high-performance, large scale, and low-cost flexible transparent electronics.

## Figures and Tables

**Table 1:** The electrical performance of IZO TFTs.

Dielectric Layer	*V*_th_ (V)	*μ* (cm^2^/Vs)	*I*_on_/*I*_off_	*SS* (V/dec.)	Anneal Temperature (°C)	Ref.
SiO_2_	2.55	0.15	2.8 × 10^6^	0.86	300	[33]
SiO_2_	-	2.3	3 × 10^4^	0.96	300	[34]
Al_2_O_3_	0.41	30.88	10^4^	-	250	[35]
Al_2_O_3_	1.32	7.23	3.05 × 10^6^	-	500	[36]
SiO_2_	-	4.94	10^7^	-	180	[31]
HfO_2_	1.1	14.3	10^7^	0.13	RT	This work

## Data Availability

Not applicable.

## References

[1] Hwang Y.H., Seo J.-S., Yun J.M., Park H., Yang S., Park S.-H.K., Bae B.-S. (2013). An ‘aqueous route’ for the fabrication of low-temperature-processable oxide flexible transparent thin-film transistors on plastic substrates. Npg Asia Mater..

[2] Liu Y., He K., Chen G., Leow W.R., Chen X. (2017). Nature-inspired structural materials for flexible electronic devices. Chem. Rev..

[3] Ahmed S., Yi J. (2017). Two-dimensional transition metal dichalcogenides and their charge carrier mobilities in field-effect transistors. Nano-Micro Lett..

[4] Liu A., Zhu H., Noh Y.-Y. (2019). Solution-processed inorganic p-channel transistors: Recent advances and perspectives. Mater. Sci. Eng. R Rep..

[5] Yang J., Zhang Y., Qin C., Ding X., Zhang J. (2019). Enhanced Stability in Zr-Doped ZnO TFTs With Minor Influence on Mobility by Atomic Layer Deposition. IEEE Trans. Electron Devices.

[6] Paterson A.F., Singh S., Fallon K.J., Hodsden T., Han Y., Schroeder B.C., Bronstein H., Heeney M., McCulloch I., Anthopoulos T.D. (2018). Recent progress in high-mobility organic transistors: A reality check. Adv. Mater..

[7] Li J., Tang W., Wang Q., Sun W., Zhang Q., Guo X., Wang X., Yan F. (2018). Solution-processable organic and hybrid gate dielectrics for printed electronics. Mater. Sci. Eng. R Rep..

[8] Park J.W., Kang B.H., Kim H.J. (2020). A review of low-temperature solution-processed metal oxide thin-film transistors for flexible electronics. Adv. Funct. Mater..

[9] Park S., Kim K.H., Jo J.W., Sung S., Kim K.T., Lee W.J., Kim J., Kim H.J., Yi G.R., Kim Y.H. (2015). In-Depth Studies on Rapid Photochemical Activation of Various Sol–Gel Metal Oxide Films for Flexible Transparent Electronics. Adv. Funct. Mater..

[10] Yang J., Wang B., Zhang Y., Ding X., Zhang J. (2018). Low-temperature combustion synthesis and UV treatment processed p-type Li: NiO x active semiconductors for high-performance electronics. J. Mater. Chem. C.

[11] Kim Y.-H., Heo J.-S., Kim T.-H., Park S., Yoon M.-H., Kim J., Oh M.S., Yi G.-R., Noh Y.-Y., Park S.K. (2012). Flexible metal-oxide devices made by room-temperature photochemical activation of sol–gel films. Nature.

[12] Wang B., Huang W., Chi L., Al-Hashimi M., Marks T.J., Facchetti A. (2018). High-k gate dielectrics for emerging flexible and stretchable electronics. Chem. Rev..

[13] Wang Z., Nayak P.K., Caraveo-Frescas J.A., Alshareef H.N. (2016). Recent Developments in p-Type Oxide Semiconductor Materials and Devices. Adv. Mater..

[14] Ţălu Ş. (2015). Micro and Nanoscale Characterization of Three Dimensional Surfaces: Basics and Applications.

[15] Mwema F.M., Akinlabi E.T., Oladijo O.P., Fatoba O.S., Akinlabi S.A., Tălu S. (2020). Advances in manufacturing analysis: Fractal theory in modern manufacturing. Modern Manufacturing Processes.

[16] Ivanda M., Musić S., Popović S., Gotić M. (1999). XRD, Raman and FT-IR spectroscopic observations of nanosized TiO_2_ synthesized by the sol–gel method based on an esterification reaction. J. Mol. Struct..

[17] Umeda K., Miyasako T., Sugiyama A., Tanaka A., Suzuki M., Tokumitsu E., Shimoda T. (2013). Impact of UV/O_3_ treatment on solution-processed amorphous InGaZnO_4_ thin-film transistors. J. Appl. Phys..

[18] Yang J., Zhang Y., Wu Q., Dussarrat C., Qi J., Zhu W., Ding X., Zhang J. (2019). High-Performance 1-V ZnO Thin-Film Transistors With Ultrathin, ALD-Processed ZrO_2_ Gate Dielectric. IEEE Trans. Electron Devices.

[19] Liu A., Liu G., Shan F., Zhu H., Xu S., Liu J., Shin B., Lee W. (2014). Room-temperature fabrication of ultra-thin ZrO_x_ dielectric for high-performance InTiZnO thin-film transistors. Curr. Appl. Phys..

[20] Lee E., Ko J., Lim K.H., Kim K., Park S.Y., Myoung J.M., Kim Y.S. (2014). Gate Capacitance-Dependent Field-Effect Mobility in Solution-Processed Oxide Semiconductor Thin-Film Transistors. Adv. Funct. Mater..

[21] Xu W., Li H., Xu J.-B., Wang L. (2018). Recent advances of solution-processed metal oxide thin-film transistors. ACS Appl. Mater. Interfaces.

[22] Zhang Y., Zhang H., Che B., Yang J., Zhang J., Ding X. (2020). A New “Ammonia Bath” Method for Realizing Nitrogen Doping in ZnSnO Transistors. IEEE Electron Device Lett..

[23] Zhang Y., Zhang H., Yang J., Ding X., Zhang J. (2019). Solution-Processed Yttrium-Doped IZTO Semiconductors for High-Stability Thin Film Transistor Applications. IEEE Trans. Electron Devices.

[24] Xu X., Cui Q., Jin Y., Guo X. (2012). Low-voltage zinc oxide thin-film transistors with solution-processed channel and dielectric layers below 150 C. Appl. Phys. Lett..

[25] John R.A., Chien N.A., Shukla S., Tiwari N., Shi C., Ing N.G., Mathews N. (2016). Low-Temperature Chemical Transformations for High-Performance Solution-Processed Oxide Transistors. Chem. Mater..

[26] Jallorina M.P.A., Bermundo J.P.S., Fujii M.N., Ishikawa Y., Uraoka Y. (2018). Significant mobility improvement of amorphous In-Ga-Zn-O thin-film transistors annealed in a low temperature wet ambient environment. Appl. Phys. Lett..

[27] Safaruddin A.S., Bermundo J.P.S., Yoshida N., Nonaka T., Fujii M.N., Ishikawa Y., Uraoka Y. (2019). Highly reliable low-temperature (180 °C) solution-processed passivation for amorphous In–Zn–O thin-film transistors. Appl. Phys. Express.

[28] Kim G.H., Jeong W.H., Du Ahn B., Shin H.S., Kim H.J., Kim H.J., Ryu M.-K., Park K.-B., Seon J.-B., Lee S.-Y. (2010). Investigation of the effects of Mg incorporation into InZnO for high-performance and high-stability solution-processed thin film transistors. Appl. Phys. Lett..

[29] Kim J.-H., Kim J., Jeong S.M., Jeong J. (2015). Storage-period dependent bias-stress instability of solution-processed amorphous indium–zinc-oxide thin-film transistors. Curr. Appl. Phys..

[30] Chiu I.-C., Cheng I.-C. (2013). Gate-bias stress stability of p-type SnO thin-film transistors fabricated by RF-sputtering. IEEE Electron Device Lett..

[31] Safaruddin A.S., Bermundo J.P.S., Yoshida N., Nonaka T., Fujii M.N., Ishikawa Y., Uraoka Y. (2020). Improvement in Bias Stress Stability of Solution-Processed Amorphous InZnO Thin-Film Transistors via Low-Temperature Photosensitive Passivation. IEEE Electron Device Lett..

[32] Liu G., Liu A., Zhu H., Shin B., Fortunato E., Martins R., Wang Y., Shan F. (2015). Low-Temperature, Nontoxic Water-Induced Metal-Oxide Thin Films and Their Application in Thin-Film Transistors. Adv. Funct. Mater..

[33] Koo C.Y., Song K., Jun T., Kim D., Jeong Y., Kim S.-H., Ha J., Moon J. (2010). Low Temperature Solution-Processed InZnO Thin-Film Transistors. J. Electrochem. Soc..

[34] Choi J., Park J., Lim K.-H., Cho N.-k., Lee J., Jeon S., Kim Y.S. (2016). Photosensitivity of InZnO thin-film transistors using a solution process. Appl. Phys. Lett..

[35] Xu W., Long M., Zhang T., Liang L., Cao H., Zhu D., Xu J.-B. (2017). Fully solution-processed metal oxide thin-film transistors via a low-temperature aqueous route. Ceram. Int..

[36] Xia W., Xia G., Tu G., Dong X., Wang S., Liu R. (2018). Sol-gel processed high-k aluminum oxide dielectric films for fully solution-processed low-voltage thin-film transistors. Ceram. Int..

